# Angle-dependent spectral reflectance material dataset based on 945 nm time-of-flight camera measurements

**DOI:** 10.1016/j.dib.2023.109031

**Published:** 2023-03-03

**Authors:** David J. Ritter, Relindis Rott, Birgit Schlager, Stefan Muckenhuber, Simon Genser, Martin Kirchengast, Marcus Hennecke

**Affiliations:** a Virtual Vehicle Research GmbH, Inffeldgasse 21a, Graz, 8010, Austria; b AVL List GmbH, Hans-List-Platz 1, Graz, 8020, Austria; c Infineon Technologies Austria AG, Babenbergerstrasse 10, Graz, 8020, Austria; d University of Graz, Heinrichstraße 36, Graz, 8010, Austria

**Keywords:** Lidar, Reflectance, Spectral, NIR, Infrared, 945nm, Material data, Time-of-flight, Angle-dependent

## Abstract

The main objective of this article is to provide angle-dependent spectral reflectance measurements of various materials in the near infrared spectrum. In contrast to already existing reflectance libraries, e.g., NASA ECOSTRESS and Aster reflectance libraries, which consider only perpendicular reflectance measurements, the presented dataset includes angular resolution of the material reflectance. To conduct the angle-dependent spectral reflectance material measurements, a new measurement device based on a 945 nm time-of-flight camera is used, which was calibrated using Lambertian targets with defined reflectance values at 10, 50, and 95%. The spectral reflectance material measurements are taken for an angle range of 0° to 80° with 10° incremental steps and stored in table format. The developed dataset is categorized with a novel material classification, divided into four different levels of detail considering material properties and distinguishing predominantly between mutually exclusive material classes (level 1) and material types (level 2). The dataset is published open access on the open repository Zenodo with record number 7467552 and version 1.0.1 [1]. Currently, the dataset contains 283 measurements and is continuously extended in new versions on Zenodo.

**Specifications Table**

**Table:** 

Subject	Material Physics
Specific subject area	Angle-dependent spectral reflectance material measurements in the near infrared spectrum at a wavelength of λ=945nm taken by a time-of-flight camera.
Type of data	Text files with two columns: reflectance $R_{\lambda }\left(\%\right)$ and incidence angle *θ* (°). Table
How the data were acquired	Data are acquired using MATLAB 2019b/2020b with a time-of-flight camera ‘FusionSens Maxx GN8-1XNBA1 60 outdoor’, an Infineon time-of-flight sensor ‘Infineon IRS1125C’ and a 2W VCSEL illumination source, mounted on an adjustable angle measurement device. The material samples are either placed within the center of the measurement device frame or the measurement device is placed centered over the material sample. The measurements are conducted with the time-of-flight camera without any pretreatment with the exception of wet surface measurements, where the samples are sprayed with tap water using a spray bottle.
Data format	Raw
Description of data collection	The intensity values of the time-of-flight camera are calibrated to Lambertian targets with defined reflectance values at 10%, 50% and 95%. From the calibration process, a relation between intensity and reflectance of the Lambertian target is derived. From the 300 frames taken by the time-of-flight camera with a resolution of 352 × 287 pixels, only the last 100 frames are considered. A line of 40 pixels perpendicular to the tilting angle of the image center at the pixel row 145 is used for evaluation and the average intensity is then used to calculate the reflectance value in the dataset.
Data source location	Institution: Virtual Vehicle Research GmbH City: Graz Country: Austria
Data accessibility	Repository name: Zenodo Data identification number: 7467552 Version: 1.0.1 Direct URL to data: https://doi.org/10.5281/zenodo.7467552
Related research article	Muckenhuber, S.; Holzer, H.; Bockaj, Z., Automotive Lidar Modelling Approach Based on Material Properties and Lidar Capabilities, Sensors 2020, 20, 3309. https://doi.org/10.3390/s20113309

**Value of the Data**

• Researchers interested in spectral reflectance values of materials may benefit from the data by using them as a reference or extension. In particular, in the field of automotive research, a significant benefit is present as the measurements are taken with a TOF sensor acting at a wavelength range similar to most common automotive lidar sensors.
• The data may contribute to the development of physically realistic sensor models including material reflectance values e.g., ray tracing models [2].
• In contrast to the NASA ECOSTRESS library with only incident angle at surface normal, the benefit of the data is a collection of material reflectance values at a specific wavelength of λ=945nm with angular resolution between 0° to 80° in 10° incremental steps and may provide a basic reference for such angle-dependent spectral reflectance measurements.
• The dataset contains reflectance values of materials which cannot be brought into a laboratory for conducting the measurement.

## 1 Objective

A challenging task for lidar models is the realistic representation of material properties of surrounding surfaces. An essential material property is the incidence angle dependent spectral reflectance of the illuminated surface in the near-infrared spectrum. To support lidar models, a new measurement device based on a 945 nm time-of-flight camera is introduced, to conduct angle-dependent spectral reflectance measurements.

This article further elaborates the data acquisition and evaluation process. It introduces a more detailed and novel material classification compared to the originally proposed approach presented [3], and publicly provides the generated measurement dataset.

## 2 Data Description

The data presented in this article contain angle-dependent spectral reflectance material measurements at a wavelength of λ=945nm. The data are recorded for an incidence angle range of 0° to 80° with incremental steps of 10°. The measurement setup with time-of-flight camera, where the incident angle angle θ ist adjusted on both sides manually and a data recording computer is shown in Fig. 2 and is described in Section 2.1.

The measurement data of the material samples are stored in text files (.txt) with two columns: incidence angle θ and spectral reflectance value $R_{\lambda }$. As an example, the data of the material sample called *“fabric_cotton_white_1”* is shown in Table 1. This simple way of storing the measurement data enables a wide compatibility range due to very basic requirements. Therefore, the data can be read and processed not only with advanced but also with basic software. The intensity and distance pictures of the sample *“fabric_cotton_white_1”* at incidence angle θ=70° taken with the measurement device are shown in Figs. 3 and 4 both including a color scale showing the intensity and distance values, respectively.

**Table 1:** Exemplary content of the spectral reflectance material measurement data file for the material sample called *"fabric_cotton_white_1"*. The text file contains two columns: Incidence angle θ and the reflectance $R_{\lambda }$.

Incidence angle θ (°)	Reflectance $R_{\lambda }$ (%)
80	16.99149
70	26.67892
60	38.928
50	51.29004
40	61.92666
30	72.27506
20	80.02045
10	84.91023
0	87.49143

### 2.1 Material classification

**Fig. 1:**
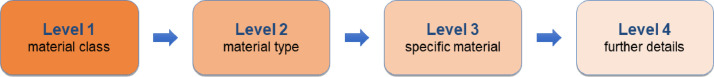
Material classification tree divided into four different levels with consecutively more details of the material.

A novel material classification scheme is introduced providing a clear distinction between the material samples. This classification is divided into four different levels, which consecutively go into more details of the material, see Fig. 1.

The first level, the material class, is chosen to be mutually exclusive which allows immediate classification and integration of newly measured materials into the material dataset. The second level, the material type, contains a subset of the material class. The specific material level three describes the subset with certain specification and level four provides further details which are applicable to many materials e.g., colors. Level one needs to be chosen, level two should be chosen, whereas level three and four are optional. However, the more information is given, the better the distinction can be made. In contrast to level 1 (mutually exclusive), any other level is extensible with new entries. Furthermore, a classification like this, where the focus lies on the material property itself, enables better reusability and allows usage for any sensor type. At the time of publication, 283 material measurements have been conducted and integrated into the dataset which include nine different material classes (level 1) and more than 30 different material types (level 2). A big part of the measurements is conducted in wet conditions, for which the material sample is either sprayed wet with a spray bottle filled with tap water or already in wet conditions due to rain.

In Table 2, an overview of the existing measurements in the dataset for the material class and material type levels is given. With the classification scheme described above, the materials are then named accordingly e.g., *“wood_timber_fir_veneered_1” (<level1>_<level2>_<level3>_level4>_<NR>)*. The previously mentioned example in Table 1 shows that certain levels may be omitted: *“fabric_cotton_white_1”* (<*level1>_<level2>_<level4>_<NR>*).

**Table 2:** Existing angle-dependent spectral material reflectance measurements within the material class and type levels in the dataset.

Level 1 (material class)	Level 2 (material type)
metal	aluminum
	iron
	steelplate

wood	cardboard
	cork
	paper
	timber

vegetation	lawn
	plant
	tree

polymer	pvc
	pu
	retroreflecting sheet
	rubber
	xps / eps

glass	tempered

fabric	cotton
	leather
	synthetic
stone	ceramic
	cobblestone
	concrete
	flagstone
	rock

soil	asphalt
	dirt / slit / mud
	gravel
	sand

cover	marking
	paint
	snow
	varnish
	water

## 3 Experimental Design, Materials and Methods

### 3.1 Measurement Device

An Infineon IRS1125C TOF sensor is cased in a ‘FusionSens Maxx GN8-1XNBA1 60 outdoor’ time-of-flight camera. This time-of-flight camera is mounted on a specific metal frame to facilitate manual adjustment of the incident angle. The camera offers different predefined use cases which differ by means of *frames per second* (FPS) and *exposure time*. Another crucial setting is the number of (recorded) frames as it must be ensured that both laser source and receiver are in thermal steady-state for proper data acquisition.

**Fig. 2:**
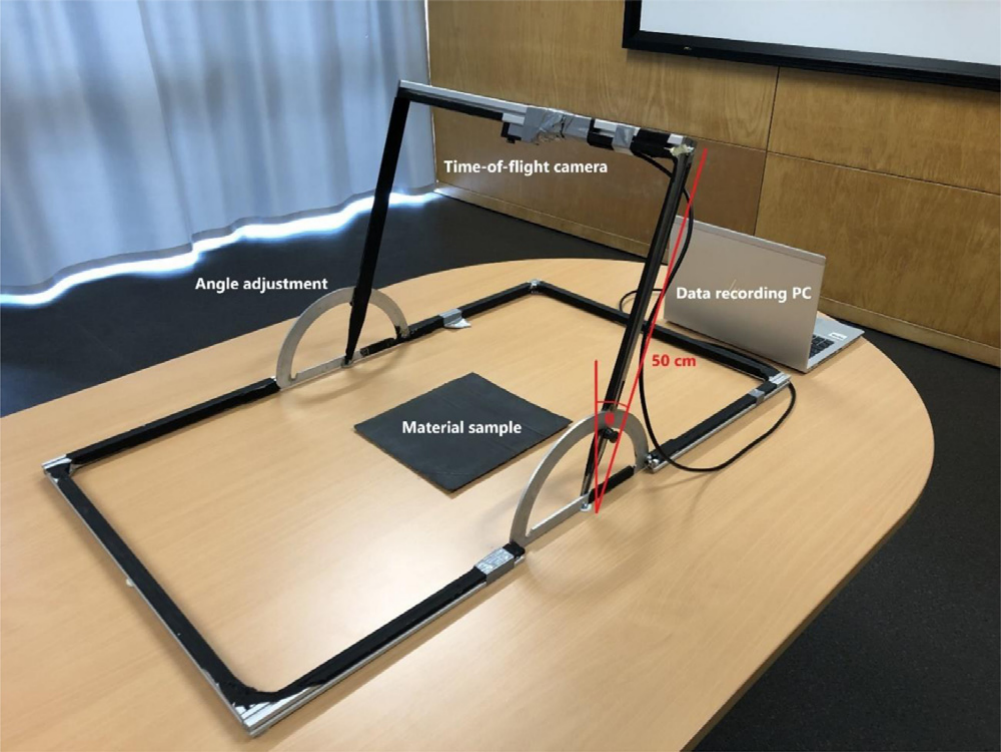
Measurement setup with time-of-flight camera and a data recording computer. The incident angle *θ* ist adjusted on both sides manually.

The data are acquired by using MATLAB scripts to communicate with the time-of-flight camera with MATLAB version 2019b and 2020b. Therefore, a framework in terms of a MATLAB wrapper called *libroyale* is needed. The measurement device is connected to the computer with a USB 3.0 cable for data recording.

### 3.2 Measurement Execution Process

1. Initialize the Camera
  Before conducting a measurement, the time-of-flight camera needs to be initialized into the MATLAB workspace which after successful creation of a camera device object is named *cameraDevice*.
2. Create a Sample
  A new sample is created with a subsequent choice of a unique sample name. This sample name is determined by the material classification in Section 1.1 and will be used for every folder and file which is related to the material sample. An additional sample description should be given as detailed as possible to provide an accurate characterization of the measurement process including sample specifics and the measurement environment.
3. Execute Measurements
  Either the material sample is placed within the center of the device frame, or the device frame is placed over the material sample in such a way that the area of interest is centered within for the data generation of the sample in Section 2.4 accordingly. To execute a measurement, the function
execute_measurement(cameraDevice, sample_name, angle)
  has to be called in the MATLAB console. The parameters include the *camera device* object which is available in the workspace from initializing the camera, the exact sample name, and the current angle of the measurement device. After successful completion of the measurement, the angle is adjusted, and the same function is called again with the new angle setting. This process is repeated until the entire angle range is recorded.

### 3.3 Measurement Device Calibration

The measurement device is calibrated by three Lambertian targets with defined reflectance values at 10%, 50%, and 95%. The time-of-flight camera measures the intensity I of the reflected light from the sample surface. This intensity value I decreases approximately according to cos(θ) which is expected to behave according to Lambert's cosine law. Based on the calibration measurements done by [3] Muckenhuber et al., a relation between the intensity I and the spectral reflectance value $R_{\lambda }\left(\%\right)$ of a Lambertian target at incidence angle $\theta =0^{\circ }$ is expressed as:

(1) $$R_{\lambda }\left(\%\right)=-0.2130+0.0698\;\times I$$

The corresponding spectral reflectance for the intensity values collected by the time-of-flight camera for a material at incidence angles θ are calculated by using Eq. (1). During the calibration process, it was observed that the camera needs up to 150 frames to be in a thermal steady-state. Therefore, 300 frames are be taken for each measurement.

A more detailed description of the calibration process is given in [3].

### 3.4 Measurement evaluation

**Fig. 3:**
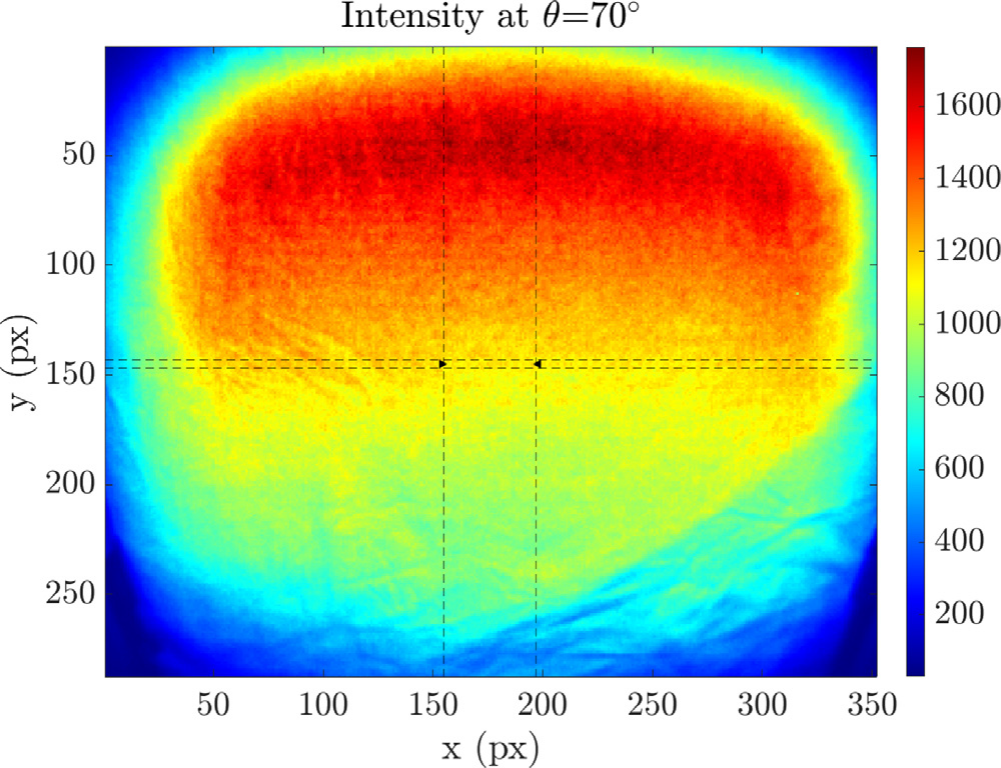
The time-of-flight camera's intensity picture of the material sample *"fabric_cotton_white_1"* at incidence angle *θ*=70°. The color scale on the right shows the intensity values whereas the left and lower axes give the pixel number.

**Fig. 4:**
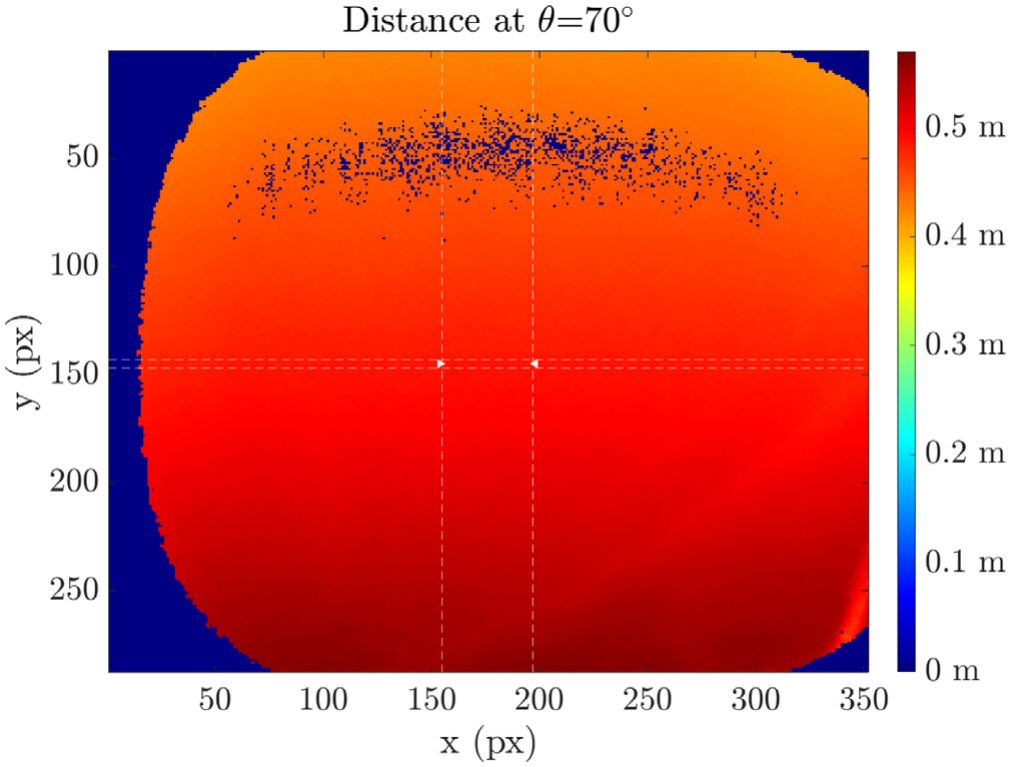
The time-of-flight camera's distance picture of the material sample *"fabric_cotton_white_1"* at incidence angle *θ*=70°. The color scale on the right shows the intensity values whereas the left and lower axes give the pixel number.

If all angle measurements for a material are finished, an evaluation script is executed yielding two images, an intensity image, and a distance image, both with color scaling. Intensity and distance data of the material *“fabric_cotton_white_1”* at $\theta =70^{\circ }$ are shown in Figs. 3 and 4, respectively. The images have a resolution of 352 × 287 pixels. Only a line of 40 pixels (pixels 156-196 out of 352) of the image center at pixel row 145 is used for evaluation. From the 300 frames taken during each measurement, 200 are dismissed and only the last 100 are used to ensure a thermal steady-state evaluation. Therefore, the resulting intensity picture shown in Fig. 3 is the average of the 40 intensity values I of the center pixels of the last 100 measurements representing 4000 single intensity measurement points. Furthermore, the text-file with two columns including incidence angle θ and spectral reflectance $R_{\lambda }$ is created with the material sample name and saved in a separate ‘database’ folder.

## Ethics Statements

This dataset generation campaign did not involve human subjects, animal experiments, or data collected from social media platforms.

## CRediT authorship contribution statement

**David J. Ritter:** Software, Validation, Writing – original draft, Investigation, Data curation, Visualization, Resources. **Relindis Rott:** Writing – review & editing. **Birgit Schlager:** Resources, Writing – review & editing. **Stefan Muckenhuber:** Methodology, Formal analysis, Writing – review & editing. **Simon Genser:** Writing – review & editing. **Martin Kirchengast:** Writing – review & editing. **Marcus Hennecke:** Writing – review & editing.

## Declaration of Competing Interest

The authors declare that they have no known competing financial interests or personal relationships that could have appeared to influence the work reported in this paper.

## Data Availability

Angle Dependent Spectral Reflectance Material Database based on 945 nm Time-of-Flight Camera Measurements (Original data) (SOL Genomics).

## References

[1] Ritter D.J. (2022). Angle dependent spectral reflectance material dataset based on 945 nm time-of-flight camera measurements. Zenodo.

[2] Rott R. (2022, pp. 1-6). Proceedings of the 2022 International Conference on Connected Vehicle and Expo (ICCVE).

[3] Muckenhuber S., Holzer H., Zrinka B. (2020). Automotive lidar modelling approach based on material properties and lidar capabilities, Sensors 20.

