# Corrigendum to “Angle-dependent spectral reflectance material dataset based on 945 nm time-of-flight camera measurements” Data in Brief 48 (2023) 109031

**DOI:** 10.1016/j.dib.2024.110337

**Published:** 2024-03-19

**Authors:** David J. Ritter, Relindis Rott, Birgit Schlager, Stefan Muckenhuber, Simon Genser, Martin Kirchengast, Marcus Hennecke

**Affiliations:** a Virtual Vehicle Research GmbH, Inffeldgasse 21a, Graz, 8010, Austria; b AVL List GmbH, Hans-List-Platz 1, Graz, 8020, Austria; c Infineon Technologies Austria AG, Babenbergerstrasse 10, Graz, 8020, Austria; d University of Graz, Heinrichstraße 36, Graz, 8010, Austria

Legend:

- Redtextisoldversion
- Greentextisnew,correctedversion
- **Bold are the mistakes**

The authors regret the following mistakes:

• In abstract and data description (page 4) section:
  ○ “...contains283measurements...'' → “...contains244measurements...''
  ○ “...,283materialmeasurements...'' → “...,244materialmeasurements...''
• In section 3.3 Measurement Device Calibration:
  ○ “...,300framesarebetaken...'' → “...300framesaretaken...''
• In specification table under “Description of data collection” and in section 3.4 Measurement evaluation:
  ○ Alineof40pixelsperpendiculartothetiltingangleoftheimagecenteratthepixelrow145isusedforevaluationandtheaverageintensityisthenusedtocalculatethereflectancevalueinthedataset.
    → “Alineof41pixelsperpendiculartothetiltingangleoftheimagecenteratpixel row145ofeachoftheframes,summinguptoatotalof4141datapointsisconsidered forevaluation.AnoutlierdetectionfunctionofMATLABwiththeGrubbs'testmethod removesoutlierdatapoints.Theaverageintensityisthenusedtocalculatethereflectance valueinthedataset.
  ○ Onlyalineof40pixels(pixels156-196outof352)oftheimagecenteratpixelrow145isusedforevaluation.Fromthe300framestakenduringeachmeasurement,200aredismissedandonlythelast100areusedtoensureathermalsteady-stateevaluation.Therefore,theresultingintensitypictureshowninFig.3istheaverageofthe40intensityvaluesIofthecenterpixelsofthelast100measurementsrepresenting4000singleintensitymeasurementpoints.
    → “Onlyalineof41pixels(pixels156-196outof352)oftheimagecenteratpixelrow 145isusedforevaluation.Fromthe300framestakenduringeachmeasurement,only thelast101framesareusedtoensureathermalsteady-stateevaluation.Therefore,the resultingintensitypictureshowninFig.3istheaverageofthe41intensityvaluesof Iofthecenterpixelsofthelast101measurementsrepresenting4141singleintensity “measurementspointsincludingtheomittedoutlierpointswhicharedetectedbythe MATLABfunction``isoutlier"withtheGrubbstestmethod.
• Fig. 3 and Fig. 4 and their descriptions:
  ○ $\text{“...}\;\text{incidence}\;\text{angle}\;\text{theta=70}{}^{\circ }\text{.''}\;$ → $\text{“...}\;\text{incidence}\;\text{angle}\;\text{theta=20}{}^{\circ }\text{.''}\;$
  ○ Thefiguresitself → “includedinthemail''

The authors would like to apologise for any inconvenience caused.

